# Insulin-mediated ciliary remodeling in the choroid plexus epithelium

**DOI:** 10.1007/s00018-026-06486-6

**Published:** 2026-10-15

**Authors:** Marleen Trapp, L. Aurica Ritter, Greta Döking, James Cleland, Anthony D. Hill, Annarita Patrizi

**Affiliations:** 1 Schaller Research Group, German Cancer Research Center (DKFZ), Heidelberg, Germany; 2 Division of Regulatory Genomics and Cancer Evolution, German Cancer Research Center (DKFZ), Heidelberg, Germany; 3 Department of Neurobiology, Interdisciplinary Centre for Neurosciences (IZN), Heidelberg University, Heidelberg, Germany; 4 Present Address: Division of Translational Pediatric Sarcoma Research and Division of Clinical Cooperation Unit Virotherapy, German Cancer Research Center (DKFZ), Heidelberg, Germany

**Keywords:** Neurodevelopment, Insulin signaling, Cilia, Intraflagellar transport, Insulin receptors, Metabolism

## Abstract

The choroid plexus (ChP) epithelium plays a crucial role in cerebrospinal fluid production and brain homeostasis. ChP is mainly composed of multiciliated epithelial cells that exhibit a noncanonical ciliary organization whose function is still debated. Insulin signaling is known to be important for central nervous system function, but its role in ChP function and ciliary organization has not been elucidated yet. To investigate how prolonged insulin exposure modulates ChP cells, we treated primary murine choroid plexus epithelial cells (pCPECs) with insulin for up to six days and used a combination of immunohistochemistry, RT-qPCR, RNA fluorescence in situ hybridization, single cell analysis and Western Blot to explore underlying molecular mechanisms. We demonstrated that prolonged insulin treatment significantly increased the percentage of multiciliated clustered pCPECs and their axoneme length. Prolonged insulin treatment selectively induced translocation of IFT20, a key component of the intraflagellar transport system, to the ciliary axoneme by increasing the cytoplasmatic surface area covered by IFT20. To note, the expression of insulin receptors (IRs) in ex vivo ChP epithelial cells started during late embryogenesis and localized predominantly to the apical membrane, but not in the cilium. Our study suggests that insulin signaling may promote ciliary remodeling in choroid plexus epithelial cells, most likely by modulating intraflagellar transport dynamics involving IFT20. These findings reveal a novel role of insulin signaling in ChP cilia organization and function, with potential implications for cerebrospinal fluid (CSF) regulation and neurodevelopmental processes.

**Supplementary Information:**

The online version contains supplementary material available at https://doi.org/10.1007/s00018-026-06486-6.

## Introduction

The choroid plexus (ChP) is a specialized membrane within the brain ventricles and consists predominantly of choroid plexus epithelial cells, as well as pericytes, fenestrated endothelial cells, fibroblasts and immune cells [1, 2]. While historically the ChP was regarded primarily as the producer of cerebrospinal fluid (CSF) and a static barrier, recent studies position the ChP as a communication hub at the interface between the central nervous system (CNS) and the peripheral environment. In fact, ChP can dynamically interact with the environment and rapidly respond to physiological and pathological stimuli, including neuroinflammation and systemic metabolic cues [3, 4]. For example, ChP expresses glucose transporters and leptin receptors and actively regulates CSF glucose and leptin concentration in response to blood glucose and leptin levels [5–7].

ChP is also known to produce and secrete multiple bioactive factors directly in to the CSF, including insulin [8], epidermal growth factor (EGF) [9] and fibroblast growth factor (FGF) [10] among others [11, 12]. For example, overexpression or downregulation of insulin gene 2 (*Ins2*), by adeno-associated-virus (AAV) or genetic knockout in the choroid plexus, lead to changes in hippocampal-dependent behaviors [13], demonstrating that ChP is one of the sources of insulin in the brain. It has, therefore, been suggested that ChP-derived-insulin plays a major role in modulating brain insulin signaling, affecting CNS metabolism, food intake and associated pathology’s such as Alzheimer’s [14, 15]. Consistent with this, insulin receptors (IRs) have been detected in the ChP by autoradiography [16] as well as by more recent RNA-scope approaches [17]. However, the precise distribution and developmental dynamics of IRs within the ChP, as well as their downstream signaling effects on the ChP itself, have remained largely unexplored.

ChP epithelial cells commit to a multiciliated fate as early as embryonic stage (E)10.5, shortly after exiting the cell cycle. At this point, the majority of ChP epithelial cells contain only a single cilium (sc); later, multiple scattered and disoriented cilia emerge (mcd stage), and, finally, cilia cluster in one location with parallel orientation (mcc stage) [18]. In addition, ChP cilia have an atypical conformation. In particular, murine ChP cilia are characterized by a 9 + 0 axonemal tubulin configuration with a discontinuous unknown electron-dense component in place of the central pairs, multiple basal feet and outer dynein arms, displaying structural properties of both sensory and motile cilia [18]. It was recently demonstrated that ChP cilia can act as sensory organelles, enabling them to transduce Sonic Hedgehog (Shh) signaling in whole ChP-explants as well as in vivo models. In fact, *FoxJ1* ChP epithelial cell knockout mice are characterized by shorter cilia unable to induce Smo-mediated pathway activation leading to changes in CSF production and ventricular enlargement [19]. In addition, Banizes et al. 2007 demonstrated that mice with an insertional mutation of *Ift88*, *Tg737*(orpk), lose ChP cilia which is accompanied by elevated cAMP, dysregulated Na⁺/HCO₃⁻ transport, and disturbed intracellular pH. To note, *Tg737*(orpk) mutants also develop perinatal hydrocephalus [20]. These data suggest that ChP cilia can act as sensory organelles and can drive hydrocephalus for example via altered epithelial ion and fluid transport [21, 22].

Cilia – both primary (sensory) and motile (multiciliated) - are assembled and maintained by intraflagellar transport (IFT), a conserved microtubule-based trafficking system that selects, transports, and positions ciliary components [23]. Two well-studied IFT proteins, IFT20 and IFT88, play distinct but complimentary roles across ciliary subtypes. IFT20 transports ciliary cargo from the Golgi apparatus to the ciliary base and locates predominantly to the Golgi apparatus. Short harpin RNA mediated *Ift20* knockdown in human retinal pigmented epithelium cells reduces ciliation by blocking assembly without disrupting the Golgi apparatus [24]. IFT88 mediates anterograde transport along the axoneme and localizes within the ciliary shaft [25]. Conditional *Ift88* deletion in primary ciliated neural crest cells (*Wnt1*-Cre; *Ift88*^fx/−^) ablates axonemes while preserving basal bodies [26], while ChP epithelial cell-specific *Ift88* deletion reduces both cilia number and axoneme length in multiciliated cells [19].

In this manuscript we explored the consequences of prolonged exposure to growth factors (FGF, EGF, hydrocortisone (HC), insulin (INS) and insulin like growth factor 1 (IGF-1)) on primary murine choroid plexus epithelial cells (pCPEC), focusing on cilia organization and length. Insulin had the strongest effect, altering ChP cilia organization and increasing axoneme length, whereas other growth factors showed mild or no effects. We demonstrated that prolonged insulin treatment modified the localization and surface area of IFT20 but not IFT88. To understand the ChP ability to sense insulin we characterized the developmental expression and localization of the IRs in the ChP using protein and mRNA-based approaches. Our findings demonstrated that IRs specifically localize to the apical CSF-facing side of ChP epithelial cell and their expression peaks during late embryogenesis and remains unchanged until adulthood.

## Materials & Methods

### Experimental animals

Inbred C57BL/6J and C57BL/6 N mice were purchased from Janvier lab and housed in both the Interdisciplinary Neurobehavioral Core (INBC) at Heidelberg University and the German Cancer Research Center (DKFZ) animal facility. GFP-tagged *Otx2* mouse line was kindly provided by Dr. Thomas Lamonerie [27]. Animals were housed in static cages, no environmental enrichment toys were added to the cages, only extra Kimwipes tissue. After weaning, animals were socially housed in groups of two to five animals per cage. Animals were housed in a temperature and humidity-controlled room on a 12 h light/12 h dark cycle, with food and water provided *ad libitum*. All animal experiments were approved by the German and European Union regulations and the guidelines of the local regional ethics committee (Regierungspräsidium Karlsruhe, Germany, protocol number G-114/18). Animal studies are reported in compliance with the ARRIVE guidelines. All methods were conducted following German Animal Welfare Act regulations.

### Primary choroid plexus epithelial cell culture

Preparation of pCPEC was adapted from a published protocol [28]. Shortly, postnatal day (P) 0–2 mice were sacrificed by decapitation. Lateral and fourth ventricle ChP were isolated and pooled together. Due to the small size of the neonatal tissue, samples from all pups coming from one litter (8–12 pups, males and females) were pooled for each experiment. Per condition 2–3 biological replicates were used, the specific numbers are indicated in the corresponding figure legends. ChP tissue was treated with 1 mg/mL Pronase (Sigma Aldrich, #10165921001) for 10 min at 37 °C. To remove fibroblasts, cells were allowed to attach in DMEM/F12 (Gibco, #11320033), 10% FBS (Gibco, #333a3164), 1% Penicillin/Streptomycin (Gibco, #15070063) for 3 h at 37 °C. For monolayer culture, dissociated cells were plated on laminin (Sigma Aldrich, #L2020; 1 µg/cm^2^) coated 24-well plates at 0.5 × 10^5^ cells/mL in DMEM/F12, 10% FBS, 2 mM L-Glutamine (Gibco,#25030024), 50 µg/mL gentamicin (Gibco, #15710049), 20 µM AraC (Sigma Aldrich, #C1768) and allowed to reach plate confluence (typically by 5–6 days).

### Primary choroid plexus epithelial cell culture drug treatment

pCPEC were treated with either 5 µg/mL human insulin (Sigma Aldrich, #I9278), 5 µg/mL IGF-1 (BioTechne GmbH, #291-G1-200), 10 ng/mL EGF (PeproTech GmbH, #315-09-100), 2 µg/mL HC (Sigma Aldrich, #H4001) or 5 ng/mL FGF (PeproTech GmbH, #450–33 A-10) for 6 consecutive days, since seeding. Alternatively, pCPEC were treated with 5 µg/mL INS for a shorter period: 24 h (INS 24h), 48 h (INS 48h) and immediately processed or they were allowed to recover for up to 5-day (d), 24h+5d and 48h+4d, respectively. For all conditions half of the media was changed every two days to maintain the concentration of the growth factors constant.

### Tissue processing for light microscopy

Male P60 mice were either transcardially perfused with 4% PFA (Sigma Aldrich, #P6148) in PBS 0.1 M, pH 7.4 (Gibco, #10010023) or euthanized by cervical dislocation and snap frozen on dry ice. PFA-perfused brains were post-fixed in 4% PFA for 1 h at 4 °C, cryoprotected in 10, 20 and 30% sucrose (MP Biomedicals, #19401890) in PBS at 4 °C, embedded in Tissue-Tek OCT (Tissue-Tek^®^ O.C.T. Compound, Sakura, #4583) and then frozen. Whole heads from E13.5 to E16.5 mice or isolated brains from E18.5 to P10 mice were rinsed in cold PBS, embedded in Tissue-Tek OCT and directly frozen on dry ice or immersed in 4% PFA for 2 h, followed by freezing. For the embryonic and early timepoints males and females were used.

For the identification of EdU^+^ cells we used E18.5 tissue generated in Ho et al. 2023 [18].

### Immunofluorescence staining and microscopy

pCPEC, plated at 0.2 × 10^5^ cells/mL in 8-well staining chambers (Invitrogen, #155411PK), were washed in PBS, fixed with cold 100% methanol for 5 min or with 4% PFA for 10 min, and permeabilized with 0.1% Triton X-100 (Sigma Aldrich, #T9284) in PBS for 15 min. Fixed pCPEC were blocked with 1% bovine serum albumin (AppliChem, #A65880100) and 1% milk in PBS for 1 h at room temperature (RT). Antibodies were diluted in the blocking solution. Primary antibody incubation was performed overnight at 4 °C. Secondary antibody incubation was carried out for 2 h at RT. Nuclei were stained with Hoechst 33342 (Biomol, #Cay15547-25; 1:4000) for 10 min at RT. For proliferation analysis, 10 µM EdU was added to plated pCPEC for 48 h, followed by 72 h without EdU before fixation with 4% PFA. The Click EdU Alexa 594 imaging Kit (Invitrogen, #C10339) was used according to the manufacturer’s instructions.

All brains were sliced in 15 μm-thick coronal or sagittal sections using a cryostat (Leica CM1950). Prior to immunostaining, frozen sections were fixed with either cold 4% PFA for 10 min or cold 100% methanol for 5 min on ice, followed by rinsing with PBS 0.01 M. Sections were then blocked and permeabilized with 10% normal donkey serum (Biozol Diagnostica, #LIN-END9010-10) and 0.5% Triton X-100 in PBS for 1 h at RT. Primary antibodies were incubated overnight at 4 °C, followed by a 2 h incubation with secondary antibodies at RT in antibody solution (3% normal donkey serum and 0.5% Triton X-100 in PBS). Sections were counterstained with Hoechst for 8 min at RT and mounted using Dako Fluorescent Mounting Medium (Agilent Technologies Inc, #GM304). Primary antibodies can be found in Table 1.

**Table 1:** List of primary antibodies for immunofluorescence

Target	Host species	Vendor	Cat. number	RRID	Dilution	Fixation
Zona occludens-1 (ZO-1)	Rat	Invitrogen	14–9776-82	AB_2573026	1:100	5 min Methanol/10 min PFA
Isolectin GS-IB4 Alexa Fluor 647 conjugate		Invitrogen	I32450		1:200	
γ-Tubulin	Mouse	Invitrogen	MA119421	AB_1075282	1:1000	5 min Methanol
ARL13B	Rabbit	Proteintech	17711-1-AP	AB_2060867	1:800	
Pan-cytokeratin	Mouse	Sigma-Aldrich	C2562	AB_476839	1:500	
IFT88	Rabbit	Proteintech	13967-1-AP	AB_2121979	1:200	
Insulin receptor beta subunit (IRβ)	Rabbit	Cell Signaling Technology	3025	AB_2280448	1:250	10 min PFA
E-cadherin	Mouse	Becton Dickinson	610,182	AB_397581	1:200	
α-Tubulin	Mouse	Sigma-Aldrich	T9026	AB_477593	1:1000	
GFP	Chicken	Hölzel	GFP-1010	AB_2307313	1:1000	
Aquaporin 1	Mouse	Santa Cruz Biotechnology	sc-55,466	AB_628739	1:500	
Glutamylated tubulin	Mouse	AdipoGen	AG-20B-0020	AB_2490210	1:800	
IFT20	Rabbit	Proteintech	13615-1-AP	AB_2280001	1:200	
58K Golgi protein	Mouse	Abcam	ab27043	AB_2107005	1:500	

Secondary antibodies were selected from the ones listed in Table 2.

**Table 2:** List of secondary antibodies for immunofluorescence

Target species	Host species	Fluorophore	Vendor	Cat. number	RRID	Dilution
Rabbit	Donkey	Alexa Fluor 647	Jackson ImmunoResearch	711-605-152	AB_2492288	1:500
Mouse	Donkey	Alexa Fluor 488	Jackson ImmunoResearch	715-545-150	AB_2340846	1:1250
Rat	Donkey	Alexa Fluor 647	Jackson ImmunoResearch	712-605-150	AB_2340693	1:500
Chicken	Donkey	Alexa Fluor 488	Jackson ImmunoResearch	703-545-155	AB_2340375	1:500
Rabbit	Donkey	Alexa Fluor 555	Invitrogen	A31572	AB_162543	1:800

Images were acquired using a Zeiss LSM780 or LSM900 confocal microscope with 16 bit and 2x line averaging. ZEN Black software was used for image acquisition and Fiji [29] (NIH) for processing analysis.

### Confocal image analysis

pCPEC imaging analysis was performed only on cells organized in rosette-like structures, suggesting a healthy state. 4–5 images were acquired per well.

#### pCPEC morphology and cilia analysis

For cilia analysis pCPECs were acquired with 40x objective, zoom 1, z-stacks of 0.5 μm interval and 1000 × 1000 fields. For EdU and cytokeratin analysis 20x objective, zoom 1, z-stacks of 1 μm interval and 1024 × 1024 fields were used. All images were acquired for the entire penetration of the antibodies to ensure full reconstruction of the apical surface and cilia apparatus. Analysis was performed on max-intensity projection images with Fiji [29]. The total number of EdU or cytokeratin positive (^+^) cells was manually quantified; the percentages of EdU^+^ or cytokeratin^+^ cells were calculated based on the total number of Hoechst cells; the apical surface cell area was calculated by manually drawing the outline of the apical cell border following zonula occludens-1 (ZO-1) staining; the nuclear surface area was calculated by a macro provided by Dr. Damir Krunic, which segmented and measured the outline of Hoechst^+^ structure; cilia density and number of axonemes were quantified using Cell Counter plug-in with Fiji; axoneme length was measured by drawing a line along ARL13B staining. Unless otherwise indicated, data are shown as fold change, average treated cell values divided by untreated cell (DMEM) or chronic insulin (c-INS) values.

#### Density Otx2^+^ cells

For Otx2 cell density, 2048 × 2048 fields of ChP were imaged using a 40x objective. The total number of Otx2^+^ cells was quantified at each time point and the percentage of Otx2^+^ cells was calculated based on total number of Hoechst cells.

#### Density IFT88^+^ cilia

For IFT88^+^ cilia analysis, 2048 × 2048 images, z-stack 0.5 μm were acquired using a 40x objective. Number of cilia and IFT88^+^ cilia where manually counted using Cell Counter plug-in with Fiji. The percentage of IFT88^+^ cilia was calculated based on the total number of cilia.

#### Colocalization IFT20 and IFT88 in cilia

For IFT20 and IFT88 cilia colocalization analysis, 2048 × 2048 images, z-stack 0.5 μm were acquired using a 40x objective. The images were processed with the image-analysis program Imaris (release 8.0.2; Bitplane). IFT20 or IFT88 colocalization with glutamylated tubulin (GlutTub^+^) cilium was estimated on segmented images with the Imaris co-localization module followed by quantification with Fiji.

#### IFT20 and Golgi apparatus surface area

For IFT20 and Golgi apparatus surface area analysis, 2048 × 2048 images, z-stack 0.5 μm were acquired using a 40x objective. The surface area covered by Golgi apparatus or by IFT20 was calculated employing a dedicated macro developed by Dr. Damir Krunic with Fiji. In particular, the macro segmented the images using a threshold that maximized the selection of immunofluorescent staining over background labeling and then measured the surface area covered by each channel. The average surface area covered by channels was subsequently plotted for each image.

### RNA fluorescence in situ hybridization (FISH) and immunofluorescence

Hybridisation chain reaction RNA FISH was performed according to the manufacturer’s instructions with minor adjustment [30] and using commercially available reagents, including riboprobes (Molecular Instruments). Cryosections from E18.5 whole heads embryos (males and females) were fixed in pre-chilled 4% PFA (Thermo Fisher Scientific, # 28908) for 15 min on ice, dehydrated through a 50–100% ethanol series at RT, rinsed with PBS, incubated in commercial probe hybridization buffer for 15 min at 37˚C, then incubated in probe solution (hybridization buffer supplemented with 4 nM *Insr* riboprobes) overnight at 37°C. The next day, sections were incubated for 15 min at 37°C in a graded wash buffer series (75% wash buffer/25% 5x SSCT, 50% wash buffer/50% 5x SSCT, 25% wash buffer/75% 5x SSCT, 0% wash buffer/100% 5x SSCT), 5 min at RT in 5x SSCT, 30 min at RT in commercial amplification buffer, then overnight at RT in hairpin solution (amplification buffer supplemented with 60 nM B2-546-h1 and B2-546-h2 hairpins). The next day, sections were incubated 3 × 20 min at RT in 5x SSCT and subjected to post RNA fluorescence in situ hybridization immunofluorescence. Briefly, sections were incubated in blocking solution (1% bovine serum albumin and 5% normal donkey serum diluted in PBST) at RT for 1 h, primary antibody solution (blocking solution supplemented with 1:200 mouse anti-E-cadherin antibody and 1:200 ALEXA Fluor 647 isolectin GS-IB_4_ conjugate) overnight at 4°C, 2 × 5 min at RT in PBST, secondary antibody solution (blocking solution supplemented with anti-mouse Alexa Fluor 647 and 1:2000 Hoechst) and 2 × 5 min at RT in PBST. Sections were mounted in Prolong Diamond, cured at RT for at least 24 h then imaged with 1536 × 1536 images, z-stack 0.5 μm using a 40x objective.

### Immunoblotting

The starting material was either cell pellets or merged snap-frozen ChP tissue. pCPECs, detached with TrypLE (Gibco, #12604013) for 20 min at 37 °C, from three 24-well were pooled for each cell pellet replicate. Lateral and fourth ChP from two P60 males or three to four P0-2 mice of mixed sex were pooled for each biological replicate. Three biological replicates were used per experiment. Tissue was lysed in ice-cold lysis buffer containing 1x RIPA-Buffer (Cell Signaling, #9806) as well as protease (Invitrogen, #A32955) and phosphatase (Invitrogen, #A32957) inhibitors. Tissue was mechanically dissolved by scratching and freeze/thaw cycles. Protein concentration was measured with BCA assay kit (Invitrogen, #23225). 30 µg of proteins were loaded on homemade 8% gels. Electrophoresis running buffer contained 2.5 mM Tris (Sigma Aldrich, #T1503), 19.2 mM Glycine (Sigma Aldrich, #13073) and 2% SDS (Carl Roth #CN30.3). Blotting was carried out using 0.45 μm PVDF membrane (Thermo Fisher Scientific, #88518) in a buffer comprised of 2.5 mM Tris, 19.2 mM Glycine and 20% Methanol (Sigma Aldrich, #106009). Membranes were blocked for 1 h at RT in 5% milk/TBS-T (Carl Roth, #1061.1). Primary and secondary antibodies were diluted in blocking buffer. Antibodies were detected using Chemiluminescence with HRP substrates Pierce ECL Substrate (Invitrogen, #32109) or WesternBright Chemiluminescence Substrate (Biozym, #541020). Images were acquired with the ChemiDoc Touch imaging system (BioRad) operated by Image Lab (v3.0.1.14). Primary and secondary antibodies can be found in Table 3.

**Table 3:** List of primary and secondary antibodies for immunoblotting

Target	Host species	Vendor	Cat. number	RRID	Dilution
Insulin receptor beta subunit (IRβ)	Rabbit	Cell Signaling Technology	3025	AB_2280448	1:250
Calnexin (CLX)	Rabbit	Abcam	ab22595	AB_2069006	1:5000
Anti mouse IgG (HRP)	Rabbit	Abcam	ab6728	AB_955440	1:5000
Anti rabbit IgG (HRP)	Goat	Abcam	ab6721	AB_955447	1:5000

### RNA isolation and reverse transcription quantitative polymerase chain reaction (RT-qPCR)

RNA extraction and RT-qPCR were performed following a previously described procedure [31]. Starting material was frozen pCPEC pellets (see immunoblotting for pellet harvesting) or ChP tissue. pCPEC from three 24-wells were pooled together for each biological replicate. ChP tissue from lateral and fourth ventricles from two P60 males or three to four mixed sex E18.5 or P0-2 mice were pooled for each biological replicate. Three biological replicates were used per experiment. RNA was extracted using QIAshredder columns (Qiagen, #79654) and RNeasy Mini Kit (Qiagen, #74104). Genomic DNA was removed by incubating samples with RNase-free DNase I (Qiagen, #79254) for 15 min at RT. RNA was eluted with 30 µl of RNase-free water. RNA quality and concentration were measured using Ultraviolet spectrophotometry on NanoDrop One (Thermo Fisher Scientific). RNA samples with absorbance ratios A260/A230 below 1.0 were precipitated using glycogen (Thermo Fisher Scientific, #R0551), ammonium acetate (Gibco, #AM9071) and 100% ethanol following the manufacturer’s protocol supplied with the glycogen. RNA was reversed-transcribed into cDNA using the High-Capacity RNA-to-cDNA kit (Applied Biosystems, #4387406). RT-qPCR was performed with 40 ng cDNA of each sample and 20 µM primer using PowerUp SYBR Green Master Mix (Thermo Fisher Scientific, #A25742). RT-qPCR were performed on a LightCycler 480 (Roche) and gene expression levels were normalized to the expression of two housekeeping genes *Tbp* and *Rpl13a* [31]. Amplification efficiency (E) values were calculated for each primer pair across samples and experiments and derived from non-baseline-corrected data using LinRegPCR (version 2020.0) [32] (Table 4). Primer sequences can be found in Table 4.

**Table 4:** List of primer sequences

Gene	Forward (5′–3′)	Reverse (5′–3′)	E (%)
*Tbp*	CCTTGTACCCTTCACCAATGAC	ACAGCCAAGATTCACGGTAGA	79
*Rlp13a*	AGCCTACCAGAAAGTTTGCTTAC	GCTTCTTCTTCCGATAGTGCATC	81
*Insr*	CAGCCGGATGGGCCAATGGGA	CTCGTCCGGCACGTACACAGAA	80
*Ift88*	TGAGGACGACCTTTACTCTGG	CTGCCATGACTGGTTCTCACT	80
*Ift20*	AGGCAGGGCTGCATTTTGAT	CAAGCTCAATTAGACCACCAACA	80

### Single cell RNA sequencing analysis

Murine embryonic and adult ChP single cell expression data [1] were analyzed in R using the Seurat package. Cells were clustered and identified by marker gene expression. After dimension reduction log-normalized counts of Insr expression were plotted on the resulting umap graphs.

### Statistical analysis

**Fig. 1:**
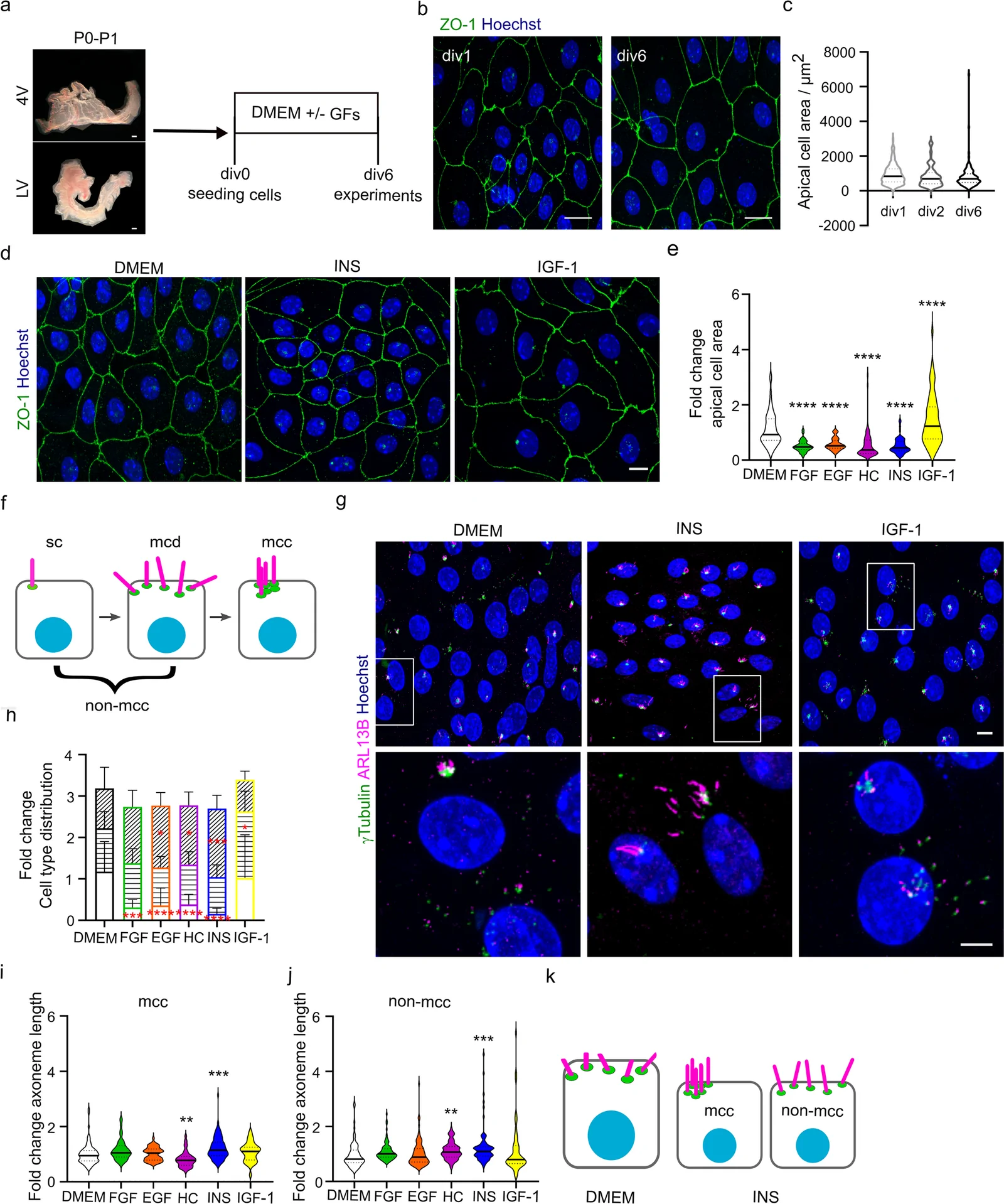
Growth factors mediate choroid plexus epithelial cell configuration. (**a**) Schematic representation of generation of primary choroid plexus epithelial cell culture (pCPEC) by combining cells from postnatal day (P) 0 or P1 lateral (LV) and fourth ventricle (4V). Experiments were performed at day *in vitro* (div) 6; (**b**) Representative confocal images of pCPEC stained against zonula occludens-1 (ZO-1) (green) at div1, 2 and 6; (**c**) Quantification of pCPEC apical cell area (µm^2^), *n* = 114–135 cells, from 3 biological replicates; (**d**) Representative confocal images of ZO-1 (green) staining of pCPEC untreated (DMEM) or treated with INS or IGF-1; (**e**) Fold change of apical cell area in pCPEC treated with FGF, EGF, HC, INS and IGF-1 compared to untreated cells, *n* = 40–60 cells, from 3 biological replicates; (**f**) Scheme of cilia types in pCPEC: non-multiciliated (non-mcc) cells combining single ciliated (sc) and multiciliated disengaged (mcd) cells and multiciliated (mcc) cells; (**g**) Representative overview images, with high magnification insets (bottom), of γ-tubulin (green) and ARL13B (magenta) staining of pCPEC untreated or treated with INS and IGF-1; (**h**) Fold change of pCPEC cell types, including non-ciliated, non-mcc and mcc, *n* = 9–15 images, from 3 biological replicates; **i**, **j**, Fold change of axoneme length of mcc cells (**i**) and of non-mcc cells (**j**), *n* = 50–80 cilia, from 3 biological replicates; **k**, Schematic representation of treatment effect on pCPEC. Scale bar 1 mm (**a**), 20 μm (**b**), 10 μm (**d**), 10 μm (**g**, top), 5 μm (**g**, bottom); Black line: median (**c**, **e**, **i**, **j**); One-way ANOVA followed by Tukey’s multiple comparisons test (**c**), Kruskal-Wallis test followed by Duns multiple comparison (**e**, **i**, **j**), Two-Way ANOVA followed by Duns multiple comparison (**h**); *p < 0.05; **p < 0.01, ***p < 0.001, ****p < 0.0001. INS, Insulin; IGF-1, Insulin-like growth factor 1; FGF, Fibroblast growth factor; EGF, Epidermal growth factor; HC, Hydrocortisone

Statistical analyses were conducted using GraphPad Prism (version 9.3.1). Due to the substantial number of animals for each pCPEC preparation we decided to reduce the number of mice used (i) by showing the drug treatment data as fold change compared to the DMEM-untreated average and (ii) by comparing shorter treatment insulin timepoints (24h, 48h, 24h+5d and 48h+4d) to the c-INS obtained in Fig. 1. These data are represented as a dashed line in Fig. 4c, d, e, h, i, j and Supplementary Fig. 2. Appropriate statistical tests were selected based on the data distribution determined with D’Agostino & Pearson test. Parametric test (One-Way ANOVA followed by Tukey’s multiple comparisons test and Two-Way ANOVA followed by Šidák`s or Tukey’s multiple comparisons test) were used only if data were normally distributed. Otherwise, nonparametric alternatives were chosen (Mann–Whitney and Kruskal–Wallis test followed by Duns multiple comparison). Data are presented as median, mean ± standard error of the mean (SEM) or mean ± standard deviation (SD). P-values in the manuscript present the following star code: *p ≤ 0.05, **p ≤ 0.01, ***p ≤ 0.001, ****p ≤ 0.0001, except for Fig. 4 and Supplementary Fig. 3 where P value is represented as ^#^*p* ≤ 0.05, ^##^*p* ≤ 0.01, ^###^*p* ≤ 0.001, ^####^*p* ≤ 0.0001 to visualize significance between short insulin treatment and c-INS. The specific statistical tests utilized, and data representation are indicated in the corresponding figure legends.

## Results

### Prolonged insulin treatment modulates choroid plexus cilia

To study the impact of prolonged exposure to well-known grown factors, such as FGF, EGF, HC, INS as well as IGF-1 [28, 33], we first established a pCPEC in vitro model of P0-1 mouse ChP (Fig. 1a). As expected, pCPECs were organized in rosette-like structures (Supplementary Fig. 1a) and more than 90% of the pCPECs were cytokeratin-positive (cyt^+^) (Supplementary Fig. 1b, c), an epithelial cell specific marker, confirming an enrichment of epithelial cells. As previously reported, pCPEC showed limited proliferation ability with less than 4% of total pCPEC to be EdU^+^ (Supplementary Fig. 1 d, e) [28]. pCPEC developed a barrier-like structure [28, 33] within 24 h from seeding (days in vitro, div1), as demonstrated by staining against ZO-1, an epithelial cell-specific tight junction protein. No clear differences was detected in the apical cell area comparing div1 to div6 pCPECs (Fig. 1b, c), suggesting that the composition of the culture media may not encourage the pCPEC to organize as cobblestone-like morphology [34]. When we exposed pCPEC to the individual growth factors we found that most of them (FGF, EGF, HC and INS) reduced the pCPEC apical cell area (0.5 ± 0.2; 0.6 ± 0.2; 0.5 ± 0.5; 0.5 ± 0.3 SD respectively) compared to the untreated control (DMEM, 1 ± 0.6 SD) (Fig. 1d, e), without affecting the total cell density or the nuclei surface area (Supplementary Fig. 1f, g) showing that an enriched culture media can induce apical constriction encouraging a cobblestone-like morphology. On the contrary, IGF-1 did not alter the apical cell area (1.5 ± 0.9 SD) (Fig. 1d, e). Of note, the concentration of the growth factor treatments was selected based on previous protocols [28, 33].

Next, we evaluated the impact of growth factors on the ciliated stage of pCPECs. ChP murine ciliogenesis starts at E10.5 and it is mainly concluded at E18.5, therefore at birth the majority of ChP epithelial cells have completed this process and appear to be mcc cells [18]. Untreated pCPEC showed three types of cilia organization: cells with a single cilium (sc cells) cells with multiple scattered and disorganized cilia (mcd), and cells with multiple clustered cilia oriented towards the CSF (mcc) (Fig. 1f; Supplementary Fig. 1 h). Up to 80% of the pCPECs were multiciliated with less than 4% ± 1.5 SEM containing a single cilium. Within the multiciliated cells, only 28% ± 5.6 SEM were mcc whereas the majority were mcd (50% ± 5.8 SEM), suggesting that in untreated conditions pCPEC cilia resemble late embryogenesis developmental stages (Supplementary Fig. 1i) [18]. Because sc accounted for less 5% of the total pCPEC and both sc and mcd represented immature cilia organizational stages [18], we grouped them together and collectively refer to them as non-mcc cells (Fig. 1f). Next, we evaluated whether growth factor treatments influence pCPEC cilia organizational stages. EGF (1.5 ± 0.3 SD), HC (1.4 ± 0.3 SD) and INS (1.7 ± 0.3 SD) significantly increased the percentage of mcc cells compared to untreated cells (1 ± 0.5 SD). This increase was most likely due to the significant reduction of the percentage of non-ciliated cells, suggesting these growth factors modulate cilia organization state. FGF significantly reduced the percentage of non-ciliated cells (Fig. 1g, h), without reaching significance for the mcc cells (1.4 ± 0.4 SD). On the contrary, IGF-1 treatment did not affect the cilia organization when compared to DMEM and the other growth factors (Fig. 1g, h). In addition, except for FGF, the growth factors, did not change the total number of axonemes per cell (Supplementary Fig. 1j, k). Interestingly, insulin treatment was enough to significantly increase the axoneme length in both mcc and non-mcc cilia, with cilia getting 2–6 times longer after insulin treatment (Fig. 1i, j). In contrast, HC treatment significantly decreased the mcc cilia length but significantly increased the non-mcc cilia length (Fig. 1i, j). To determine whether the effect on axoneme length depended on cilia maturation stage, we considered sc and mcd cilia as two independent groups. Surprisingly, only sc cilia showed a significant growth factor-dependent increase in axoneme length, except for IGF-1. Mcd cilia were not affected by any of the growth factor treatments (Supplementary Fig. 1 l). We next asked whether other parameters, such as apical cell area, were similarly influenced by cilia state. Apical cell area surface did not differ between non-ciliated and sc cells, but was significantly reduced in mcd cells upon exposure to most growth factors (Supplementary Fig. 1 m). Altogether, these data indicate that selective growth factors influence key features of pCPEC’s cilia organizational and that cilia state may in turn shape the ability of the cilium to sense specific growth factors. Notably, insulin was the only growth factor capable of modulating multiple key cilia features, ranging from cilia organizational state to axoneme length (Fig. 1k).

### Insulin receptors are enriched in choroid plexus epithelial cells from late embryogenesis

**Fig. 2:**
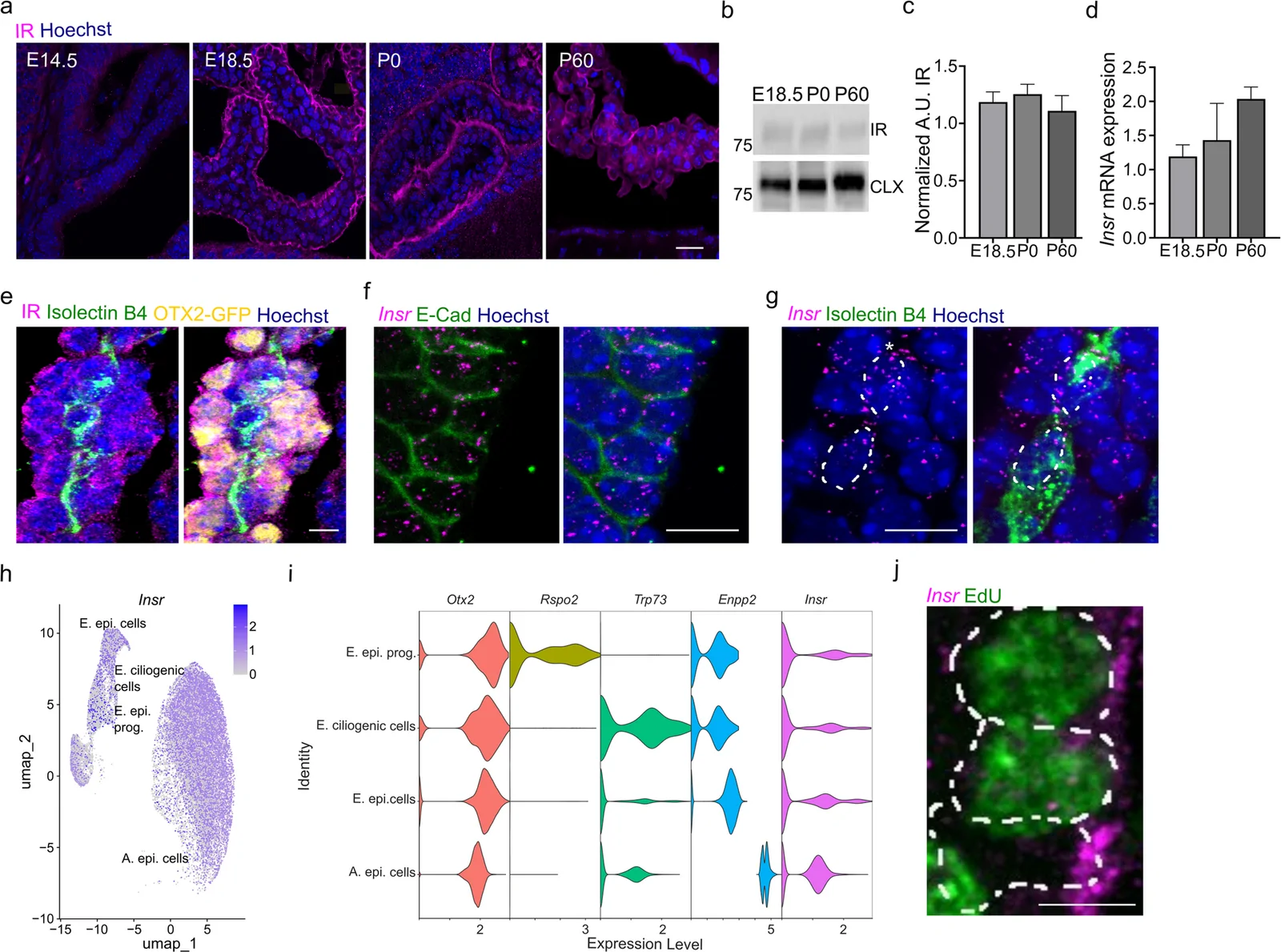
Insulin receptors are mainly expressed in choroid plexus epithelium starting at late embryogenesis. (**a**) Representative low magnification images of IR immunofluorescence (magenta) at indicated ages; (**b**) Western blot of insulin receptor (IR) and calnexin (CLX) at indicated ages; (**c**) Western blot quantification, *n* = 3 biological replicates from 2–5 mice each; (**d**) mRNA expression level of *Insr* at indicated ages, *n* = 3 biological replicates from 2–5 mice each; (**e**) Representative images of choroid plexus at embryonic day (E) 18.5 of Otx2-GFP (yellow) stained against IR (magenta) and Isolectin B4 (green); (**f**) Representative images showing *Insr* mRNA (magenta) and immunofluorescence against E-Cadherin (E-Cad, green) in the choroid plexus at E18.5; (**g**) Representative images showing *Insr* mRNA (magenta) and immunofluorescence against Isolectin B4 (green) in the choroid plexus at E18.5. (**h**) UMAP expression of *Insr* mRNA in choroid plexus epithelial cells at multiple developmental states. (**i**) Violin plot depicting expression of selected marker genes identifying choroid plexus epithelial cells at multiple developmental states. E. epi. cells, embryonic epithelial cells; E. ciliogenic cells, embryonic ciliogenic cells; E. epi. prog., embryonic epithelial progenitors; A. epi. cells, adult epithelial cells. (**j)**, Representative images of double staining against IR (magenta) and EdU (green) at E18.5. Scale bar 25 μm (**a**), 10 μm (**e**,** f**,** g**), 5 μm (**j**). Bars represent mean ± SD; Kruskal-Wallis test followed by Duns multiple comparison

To assess whether the developmental timing of ciliogenesis overlaps with the insulin pathway maturation we quantified gene and protein expression of IRs in the ChP starting at E14.5 until P60. Immunohistochemistry showed that IR protein expression was undetectable at E14.5, it peaked at E18.5 coinciding with the completion of ChP ciliogenesis [18] and it stayed stable up to P60 (Fig. 2a). Quantification of IR protein levels, from ChP homogenates, confirmed that IR expression was detectable at E18.5 and it stayed constant until P60 (Fig. 2b, c). Similarly, *Insr* mRNA expression was reliably expressed starting at E16.5 and its expression remained stable until adulthood (Fig. 2d). In the brain IRs are mainly known to be expressed in the brain capillary endothelial cells of the blood-brain-barrier [35]. To test which cell types express IRs in the ChP, we stained ChP epithelium reporter mouse, Otx2-GFP [27], against Isolectin B4 and found that the majority of Otx2^+^ cells (85% ± 2.5 SEM) were positive for IRs (Fig. 2e). Taking advantage of the spatial resolution of RNA FISH, we next confirmed that many epithelial cells expressed *Insr* mRNA (Fig. 2f), however also a subset of Isolectin B4^+^ cells expressed IRs at E18.5 (Fig. 2g). Similarly, analysis of publicly available single-nucleus RNA sequencing data from ChP at multiple developmental timepoints (embryonic and adult) confirmed that *Insr* mRNA is expressed in both epithelial and endothelial cells, with the highest expression observed in adult endothelial cells (Supplementary Fig. 2). We, next, asked whether ChP epithelial cells express *Insr* mRNA throughout all developmental stages (progenitors, multiciliated cells and differentiated/mature stages). A small but consistent subset of ChP epithelial cells expressed *Insr* mRNA at every stage examined, with peak expression in mature adult cells (Fig. 2h, i). Finally, we assessed whether actively dividing epithelial cells, identified by EdU incorporation, express IR, and found that sparse proliferative cells were already IR^+^ (Fig. 2j). All together, these findings suggest that IRs are expressed in both ChP epithelial cells and endothelial cells, starting by late embryogenesis.

**Fig. 3:**
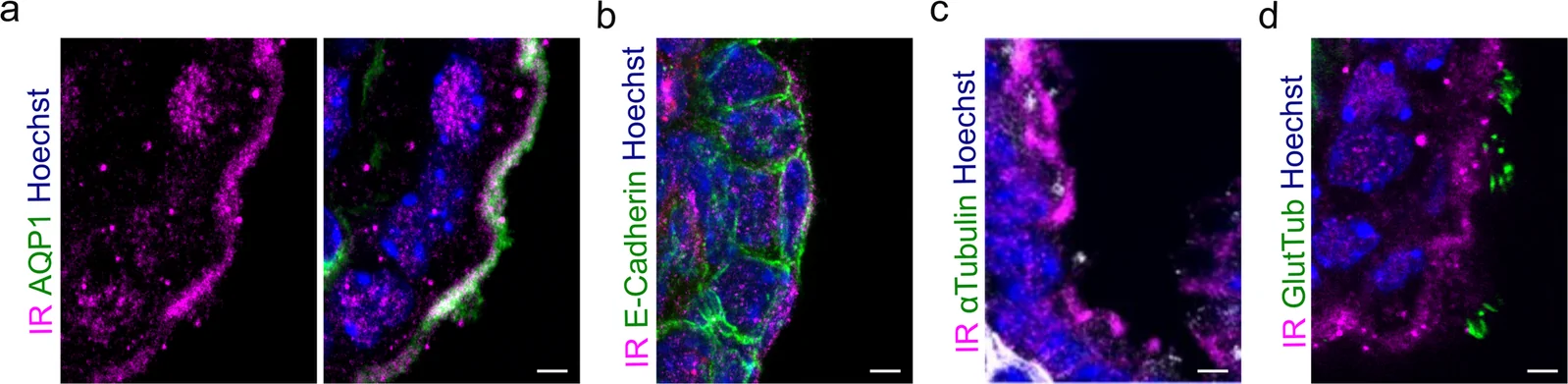
Insulin receptors locate at the apical compartment of choroid plexus epithelium but not in cilia. (**a–d**) Representative immunofluorescence images of choroid plexus epithelial cells at embryonic day (E) 18.5 stained against insulin receptors (IR, magenta) and aquaporin 1 (AQP1, green) (**a**); E-cadherin (green) (**b**); α-tubulin (green) (**c**); and glutamylated tubulin (GlutTub, green) (**d**). Scale bar 3 μm

ChP epithelial cells are polarized, with transporters strategically positioned on both apical and basal compartments, to facilitate the exchange with CSF and peripheral blood [36]. We, next, examined the subcellular localization of IRs showing that IRs selectively colocalized with aquaporin-1 (AQP1), an apical water-specific transmembrane channel [37] (Fig. 3a). Indeed, IRs did not colocalize with E-Cadherin, a marker of adherens junctions between epithelial cell-cell contact [38] (Fig. 3b), indicating IRs localized on the apical membrane facing CSF. Finally, we investigated whether ChP cilia harbor IRs. No clear localization between IRs and α-tubulin, basal body maker (Fig. 3c) or GluTub, axoneme marker, (Fig. 3d) was detected, demonstrating that IRs are not localized in the ChP nodal-like cilia.

### Insulin-induced ciliary reorganization requires prolonged exposure

**Fig. 4:**
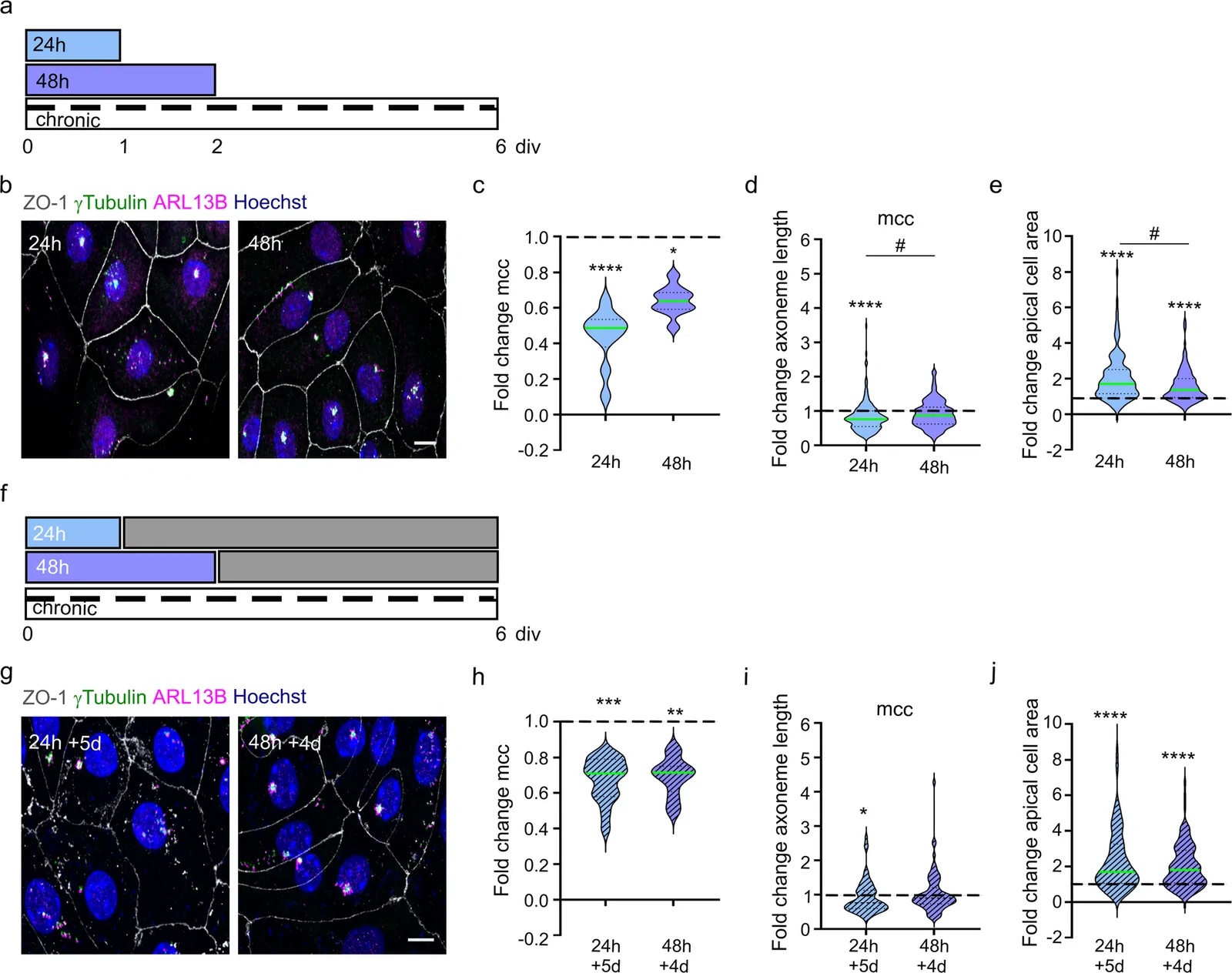
Modulation of choroid plexus cilia requires extended exposure to insulin. (**a**) Schematic representation of 24h, 48h and chronic insulin (c-INS) treatment; (**b**) Representative confocal images of primary choroid plexus epithelial cells (pCPECs) stained against zona occludens-1 (ZO-1, gray), γ-tubulin (green) and ARL13B (magenta) after 24h and 48h insulin treatment; (**c**) Fold change of the percentage (%) of multiciliated (mcc) pCPECs, *n* = 10–15 images, from 3 biological replicates; (**d**) Fold change of the axoneme length of mcc pCPEC, *n* = 70–100 cilia, from 3 biological replicates; (**e**) Fold change of the apical cell area, *n* = 115–130 cells; (**f**) Schematic representation of 24h + 5 days (d), 48h+4d and c-INS treatment; (**g**) Representative confocal images of pCPEC stained against ZO-1 (gray), γ-tubulin (green) and ARL13B (magenta) at 24h+5d and 48h+4d; (**h**) Fold change of the % of mcc pCPECs, *n* = 12–15 images, from 3 biological replicates; (**i**) Fold change of the axoneme length of pCPEC mcc, *n* = 135–180 cilia; (**j**) Fold change of the apical cell area, *n* = 110–130 cells, from 3 biological replicates. Scale bar 10 μm; Dashed line represents c-INS. Green line: median (**c**, **d**, **e**, **h**,** i**, **j**); Kruskal-Wallis test followed by Duns multiple comparison; *p < 0.05; **p < 0.01, ***p < 0.001, ****p < 0.0001, # indicates significance between c-INS and 24h, 48h, 24h+5d, 48h+4d, ^#^*p* < 0.05

To assess whether shorter insulin treatment was enough to elicit ciliary phenotypes, we treated pCPEC with insulin only for 24–48 h and compared them to pCPEC treated with insulin for 6 days (c-INS) (Fig. 4a, see statistical analysis in MM). Both shorter insulin treatments significantly decreased the percentage of mcc cells compared to c-INS (24h, 0.4 ± 0.2; 48h, 0.6 ± 0.1; c-INS, 1 ± 0.2 SD) (Fig. 4b, c). In the 24 h condition the decrease of mcc cells coincided with a significant increase in the percentage of non-mcc cells (Supplementary Fig. 3a) and an unchanged percentage of non-ciliated cells (Supplementary Fig. 3b). Whereas in the 48 h condition the decrease of mcc cells corresponded with a maintained percentage of non-mcc cells (Supplementary Fig. 3a) and the tendency to increase the percentage of non-ciliated cells (Supplementary Fig. 3b). Neither of the shorter treatment conditions affected the number of axonemes in non-mcc and mcc cells compared to c-INS (Supplementary Fig. 3c, d). Similarly, neither 24–48 h treatment affected axoneme length in non-mcc or mcc cells compared to c-INS (Supplementary Fig. 3e; Fig. 4d). In fact, after 24 h treatment mcc axoneme length was significantly shorter compared to 48 h and c-INS exposure (24h, 0.8 ± 0.4; 48h, 0.9 ± 0.4; c-INS, 1 ± 0.4 SD). Neither 24–48 h insulin treatment was enough to induce apical constriction when compared to c-INS. However, the overall apical cell area was significantly smaller at 48 h compared to 24 h insulin (24h, 2.0 ± 1.3; 48h, 1.6 ± 0.9, c-INS, 1 ± 0.6 SD) (Fig. 4e). Altogether these results revealed that prolonged exposure is necessary to influence pCPEC cilia organization, as 24 h insulin treatment resembled untreated conditions and 48 h represented an intermediate state between untreated and c-INS.

Structural ciliary reorganization may require extended time to respond to a stimulus, we, therefore, modified the treatment protocol by exposing pCPEC to insulin for 24–48 h, followed by a recovery period for up to 5-days (d), 24h+5d and 48h+4d, respectively (Fig. 4f). Neither of these conditions affected the percentage of non-mcc, non-ciliated cells (Supplementary Fig. 3f, g) and the percentage of mcc cells did not reach the level of c-INS (24h+5d, 0.7 ± 1.2; 48h+4d, 0.7 ± 0.1; c-INS, 1 ± 0.2 SD) (Fig. 4g, h). Additionally, neither of the treatment conditions affected the number of axonemes in non-mcc and mcc cells compared to c-INS (Supplementary Fig. 3 h, i). 24h+5d and 48h+4d significantly decreased the axoneme length of the non-mcc cells (Supplementary Fig. 3j) and mcc cells (Fig. 4i) compared to c-INS. Neither treatment was enough to induce apical constriction compared to c-INS (Fig. 4j). All together, these findings suggest that ciliary reorganization is time-dependent, and it requires prolonged exposure to insulin.

### Insulin treatment increases IFT20 trafficking between the Golgi apparatus and the cilium axoneme

**Fig. 5:**
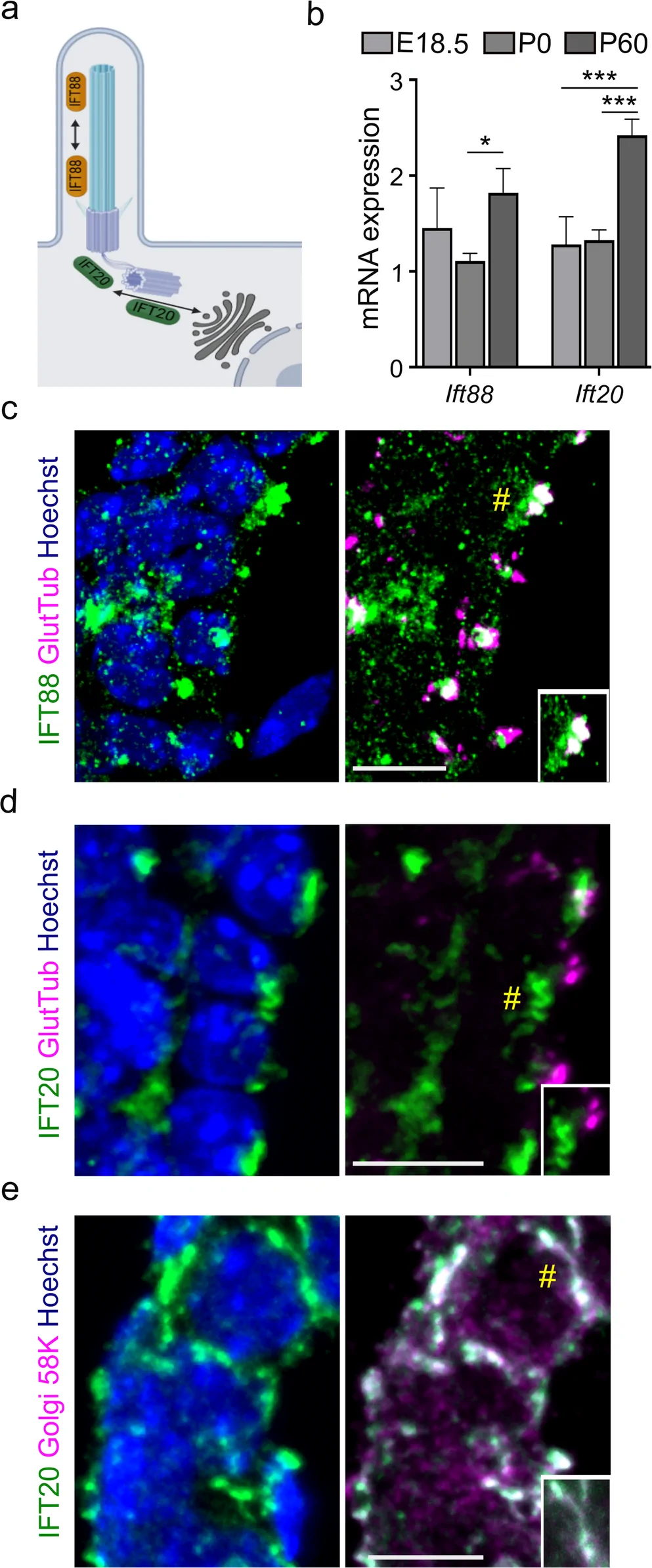
Intraflagellar transport proteins localization in choroid plexus epithelial cells. (**a**) Scheme of Intraflagellar transporter 88 and 20 (IFT88; IFT20) location (created with BioRender.com); (**b**) mRNA expression level of *Ift88* and *Ift20* at embryonic day (E) 18.5, postnatal (P) 0 and P60 mouse choroid plexus, *n* = 3 mice per age; (**c-e**) Representative images, with high magnification insets (#), of E18.5 ChP stained against IFT88 (green) and GlutTub (magenta) (**c**); IFT20 (green) and GlutTub (magenta) (**d**); and IFT20 (green) and Golgi 58K (magenta) (**e**). Scale bar 10 μm; Data are represented as mean ± SD; Two-Way ANOVA followed by Šidák`s multiple comparisons test; *p < 0.05; ***p < 0.001

To investigate the potential molecular machinery underlying how insulin modulates the cilia phenotype in the ChP, we focused on IFT88 and IFT20, proteins well-known to play a crucial role in cilia biogenesis [24, 25] (Fig. 5a). First, we investigated whether ChP expresses *Ift88* and *Ift20* by quantifying their gene expression levels from ChP homogenates. Both *Ift88* and *Ift20* mRNA were expressed already at E18.5 and their expression remained stable until P60 (Fig. 5b). Next, we used immunostaining against IFT88 and GlutTub demonstrating that IFT88 was expressed in ChP epithelial cells and that the majority of GlutTub^+^ axoneme were also IFT88^+^ (81% ± 3.4 at E18.5; 74% ± 11.6 SEM at P60), indicating that nodal-like ChP cilia contain IFT88 (Fig. 5c). Immunostaining against IFT20 and GlutTub demonstrated that IFT20 was also expressed in ChP epithelial cells, but not in GlutTub^+^ structures (Fig. 5d). Because it is known that IFT20 controls the transport of ciliary microtubules between the Golgi apparatus and the cilium [24], we next stained against Golgi protein 58K, a marker for the Golgi apparatus, confirming that IFT20 was preferentially enriched in this organelle starting at E18.5 (Fig. 5e).

**Fig. 6:**
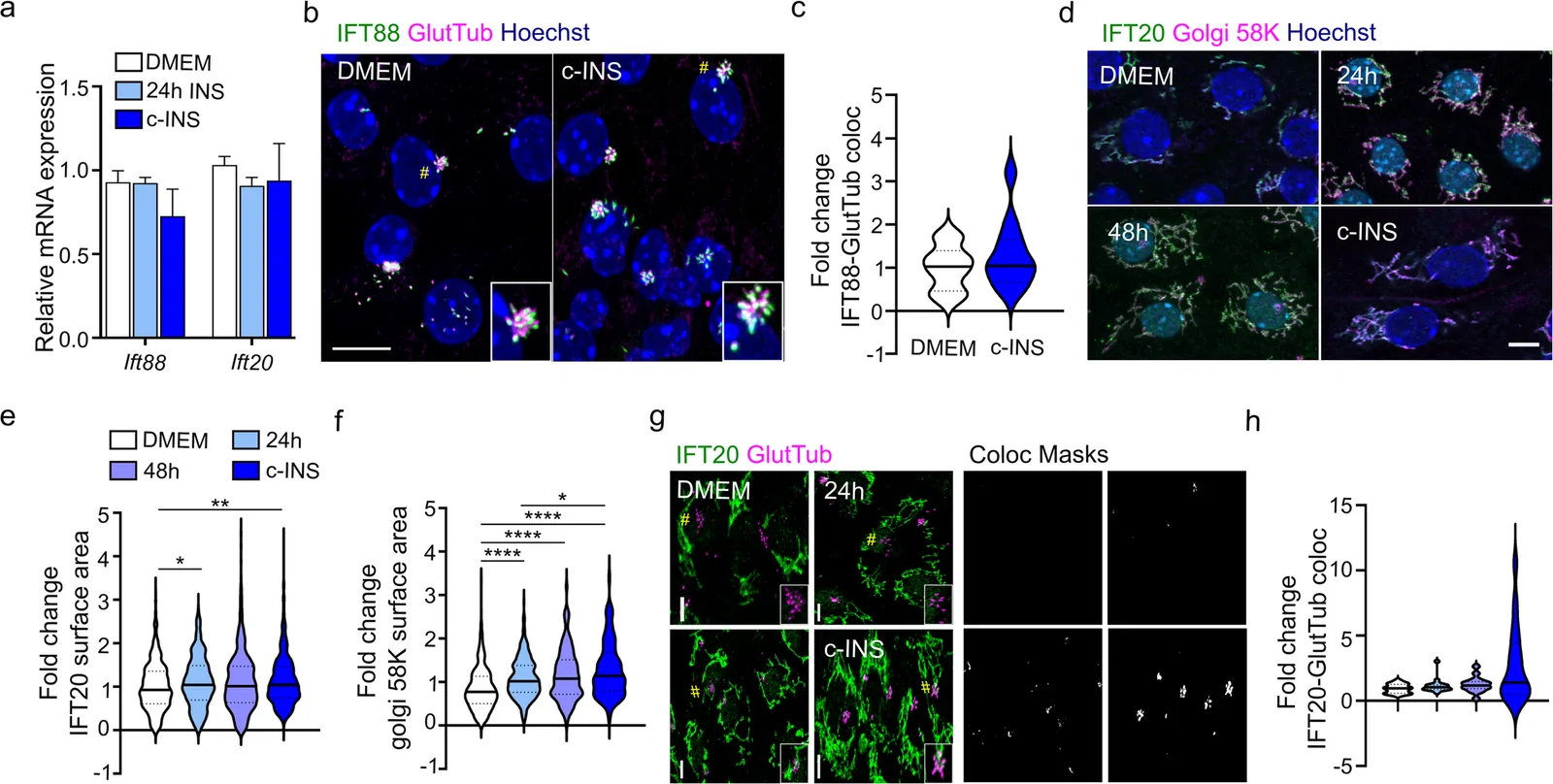
Extended exposure to insulin leads to IFT20 re-localization to the cilium apparatus. (**a**) mRNA expression levels of *Ift88* and *Ift20* in untreated (DMEM), 24 h insulin (INS) (24h), and chronic INS (c-INS) treated primary choroid plexus epithelial cells (pCPEC), *n* = 3 biological replicates; (**b**) Representative images, with high magnification insets (#), of DMEM and c-INS treated pCPEC stained against IFT88 (green) and GlutTub (magenta); (**c**) Fold change of the percentage (%) of IFT88-GlutTub colocalization signal, *n* = 10 images, from 2 biological replicates; (**d**) Representative images of DMEM, 24h, 48h and c-INS treated pCPEC stained against IFT20 (green) and Golgi 58K (magenta); (**e**) Fold change of the IFT20 surface area covered, *n* = 450–500 cells, from 3 biological replicates; (**f**) Fold change of the Golgi 58K surface area covered, *n* = 220–260 cells, from 3 biological replicates; (**g**) Representative images, with high magnification insets (#), of DMEM, 24h, 48h and c-INS treated pCPEC stained against IFT20 (green) and GlutTub (magenta). The colocalized pixels between IFT20 and GlutTub, in all treatment conditions, are shown in white (constant intensity value: 255); (**h**) Fold change of the % of IFT20-GlutTub colocalization signal, *n* = 15–20 images, from 3 biological replicates. Scale bar 10 μm; Black line: median; Two-Way ANOVA followed by Tukey’s multiple comparisons test (**a**), Mann-Whitney test (**c**), Kruskal-Wallis test followed by Duns multiple comparison (**e**,** f**,** h**); *p < 0.05; **p < 0.01, ***p < 0.001, ****p < 0.0001

Next, we evaluated whether insulin exposure can affect *Ift88* or *Ift20* gene expression levels in pCPEC at two timepoints (24h and c-INS). No significant alteration of either of the *Ift* genes was detected in any of the conditions (Fig. 6a). Similarly, the colocalization of GlutTub^+^ axoneme with IFT88 did not change after c-INS treatment (Fig. 6b, c), suggesting IFT88 is not affected by insulin treatment. On the contrary, immunostaining against IFT20 and Golgi 58K showed a significant increase in the surface area covered by both IFT20 (Fig. 6d, e) and Golgi apparatus (Fig. 6d, f) already after 24 h insulin treatment compared to untreated condition (DMEM). Finally, to evaluate whether insulin treatment can affect the IFT20 localization, we stained against GlutTub and IFT20 and quantified the colocalization of IFT20 with GlutTub^+^ axonemes. Surprisingly, we found that IFT20 can translocate to the ciliary axoneme, however only in the c-INS condition (Fig. 6g, h). All together, these findings suggest the IFT20 and Golgi apparatus can be modulated by prolonged insulin treatment resulting in a reorganization of the ciliary axoneme.

## Discussion

Our findings collectively support a model in which prolonged insulin treatment significantly increases the proportion of primary multiciliated ChP epithelial cells and promotes axonemal elongation. While FGF, EGF, and HC treatments similarly increased the percentage of multiciliated pCPECs, they did not affect ciliary length. In contrast, IGF-1 treatment exerted opposing effects on epithelial characteristics compared to the other growth factors tested. Although most of these growth factors have been detected in the CSF [39], limited data is available regarding their specific role in ChP function. Previous studies have shown that HC administration in chick embryos reduces ChP epithelial cell proliferation [40], whereas EGF, FGF, and IGF-1 promote ChP proliferation [10, 28, 41]. Our findings demonstrate that these growth factors can also influence postmitotic primary ChP epithelial cells, suggesting a role in promoting both cellular and ciliary organization beyond their established effects on proliferation. Notably, insulin has been shown to modulate intracellular Na^2+^ concentrations, thereby enhancing apical efflux of K^+^ and Cl^−^ and subsequently driving osmotic water secretion [42], indicating that insulin-mediated effects on ChP may extend to functional aspects of CSF production.

Receptors for insulin, IGF-1, EGF and FGF are known to localize to primary cilia in various cell types [43, 44], and have been implicated in regulating ciliary length through both elongation and resorption mechanisms [45, 46]. Interestingly, insulin has also been shown to act synergistically with IGF-1, EGF, and FGF. Notably, all these growth factors converge on shared downstream pathways, such as the PI3K/AKT/mTOR signaling axis, which is known to regulate primary cilia assembly and resorption [43, 47]. However, their individual or combined potential localization and functional role in ChP cilia remain uncharacterized. Our study shows that prolonged insulin treatment uniquely promotes ChP nodal-like cilia elongation, despite the absence of detectable IRs within the ciliary compartment itself. Notably, in pancreatic islets, IRs are recruited to primary cilia upon glucose stimulation [48], where ciliary motility is required for glucose-stimulated calcium influx and insulin release. Pancreatic islet cilia exhibit motility characterized by a combination of planar beating and rotational movement resembling nodal cilia, attributed to their atypical “8 + 1” microtubule configuration [49]. Similarly, ChP cilia possess a non-canonical nodal-like 9 + 0 microtubule architecture and display hybrid characteristics of both primary and motile cilia [18]. The development of purified primary ChP cell culture systems combined with high-resolution multimodal imaging technologies [50], now provides the opportunity to address several critical questions, such as whether IRs translocate to ChP cilia under specific conditions, whether insulin or other growth factors induce ChP ciliary motility, and how insulin influences the three-dimensional ultrastructural organization of ciliary microtubules and motor protein complexes.

Our study reveals that insulin and IGF-1 elicit distinct responses in pCPECs, a finding that is particularly striking given the overlapping receptor specificity of these ligands. While insulin and IGF-1 preferentially bind to their cognate tyrosine kinase receptors (IR and IGF-1R, respectively), each can cross-activate the alternate receptor with lower affinity, and IR and IGF-1R can heterodimerize to form functional hybrid receptors [15, 51]. Despite this receptor promiscuity, insulin predominantly mediates cellular metabolic responses, whereas IGF-1 primarily promotes cellular growth and proliferation [15, 52, 53]. Our data demonstrate that ex vivo IR expression peaks during late embryogenesis, coinciding with the cessation of ChP epithelial cell proliferation and full differentiation [18]. This temporal pattern suggests that insulin signaling may not be essential for ChP proliferation and early development but rather plays a role in post-mitotic cellular maturation. The insulin-dependent ciliary remodeling observed in pCPECs may therefore reflect metabolic adaptation rather than proliferative signaling. Supporting this interpretation, prolonged insulin exposure significantly increased both Golgi apparatus surface area and IFT20 distribution, suggesting enhanced protein processing and production through the secretory pathway [54, 55] and increased trafficking of ciliary components to support axonemal elongation [23]. Collectively, these findings indicate that insulin promotes metabolic adaptation of differentiated ChP epithelial cells, which manifests as enhanced ciliary growth and potentially augmented secretory capacity.

One limitation of this study is that most of our findings are based primarily on immunofluorescence analyses following prolonged insulin treatment, which does not directly establish whether insulin-IR signaling regulates ciliary structural changes or whether these represent secondary downstream effects. While our data demonstrate correlations between insulin treatment and increased multiciliation, axonemal elongation and IFT20 redistribution, the precise mechanisms remain to be elucidated. Future mechanistic validation, through loss-of-function approaches (siRNA/shRNA knockdown or CRISPR-Cas9) knockout of IR and downstream effectors such as PI3K/AKT or MAPK/ERK components [56, 57] and gain-of-function experiments (IR overexpression) will be essential to establish causality.

In the brain, IRs are expressed by capillary endothelial cells of the blood-brain-barrier, where they mediate active insulin transport from the circulation into the brain parenchyma [58]. Recent work from the Egan group demonstrate that ChP epithelial cells represent an additional source of central insulin, with local insulin secretion into the CSF occurring independently of glucose and instead being activated by serotonin [8]. Furthermore, AAV-mediated manipulation of ChP insulin expression has revealed functional consequences for brain physiology: insulin overexpression reduces anxiety-like behavior, while insulin downregulation influences feeding behavior via hypothalamic neuronal circuits [13]. Our findings demonstrate that multiciliated ChP epithelial cells sense and respond to prolonged exposure and elevated insulin concentrations. The insulin-induced ciliary remodeling observed in our study raises the possibility that ChP cilia may function as specialized sensors of local insulin levels. Future studies should determine whether endogenous insulin production engages autocrine or paracrine mechanisms which contribute to the regulation of ciliary homeostasis.

Our experimental approach also warrants some methodological considerations. First, we treated murine cells with human insulin, therefore potential species-specific differences in the binding kinetics between these two species should be considered in the future. However, insulin receptors are highly conserved across mammalian species (> 95% amino acid identity between humans and mice) [59]. Secondly, we used a supraphysiological insulin concentration in our experimental setup. Under physiological condition, CSF insulin levels are markedly lower than the plasma concentration, with reported values of approximately 0.9 ± 0.1 µU/ml in the CSF compared to 12 ± 1.2 µU/ml in circulation in humans [60, 61]. Notably, it has been suggested that the brain protects itself from hyperinsulinemia, as multiple studies have demonstrated reduced CSF insulin levels in diabetic and obese patients due to decreased IR binding affinity and consequent impairment of IR-mediated insulin transport across the blood-brain barrier [62–64]. Interestingly, alterations in primary neuronal cilia have been observed in response to high fat diet and impaired insulin signaling; however, these changes – characterized by ciliary shortening and decreased cilia numbers [65] – are opposite to the insulin induced ciliary elongation observed in our ChP model. These contrasting findings suggest that metabolic dysregulation may influence ciliary remodeling in a cell type specific way, with neurons and ChP epithelial cells responding differently to alterations in insulin signaling. Future studies examining ChP cilia in models of metabolic disease will be critical to understanding the physiological relevance of insulin-mediated ciliary remodeling and its contribution to metabolic dysfunction in the brain barriers.

## Conclusion

Beyond its role in CSF production, the ChP can sense and respond to multiple growth factors shaping key differentiation hallmarks, such as cell and cilia organization. Given that insulin receptor resistance in the brain correlates with some brain diseases, our study supports the need to uncover the role of cilia in the insulin signaling pathway in the ChP.

## Supplementary Information

Below is the link to the electronic supplementary material.

Supplementary Material 1

## Data Availability

All data generated during this study are included in this published article. The developed analysis tools are available from the corresponding author upon request.

## References

[1] Dani N, Herbst RH, McCabe C, Green GS, Kaiser K, Head JP et al (2021) A cellular and spatial map of the choroid plexus across brain ventricles and ages. Cell 184(11):3056–3074e21. 10.1016/j.cell.2021.04.003 PubMed PMID: 33932339; PubMed Central PMCID: PMC8214809 PMC8214809 33932339

[2] Hill AD, Okonechnikov K, Herr MK, Thomas C, Thongjuea S, Hasselblatt M et al (2024) Single-nucleus RNA-seq dissection of choroid plexus tumor cell heterogeneity. EMBO J 43(24):6766–6791. 10.1038/s44318-024-00283-2 PubMed PMID: 39482394; PubMed Central PMCID: PMC11649822 PMC11649822 39482394

[3] Scarpetta V, Ho KH, Trapp M, Patrizi A (2025) Choroid plexus: Insights from distinct epithelial cellular components. Curr Opin Neurobiol 93:103028. 10.1016/j.conb.2025.103028 PubMed PMID: 40267629 40267629

[4] Courtney Y, Hochstetler A, Lehtinen MK (2025) Choroid Plexus Pathophysiology. Annu Rev Pathol 20(1):193–220. 10.1146/annurev-pathmechdis-051222-114051 PubMed PMID: 39383438; PubMed Central PMCID: PMC11884907 PMC11884907 39383438

[5] Banks WA, Kastin AJ, Huang W, Jaspan JB, Maness LM (1996) Leptin enters the brain by a saturable system independent of insulin. Peptides 17(2):305–311. 10.1016/0196-9781(96)00025-3 PubMed PMID: 8801538 8801538

[6] Vernau W, Vernau KA, Sue Bailey C (2008) Cerebrospinal fluid. Clinical biochemistry of domestic animals. Elsevier, pp 769–819. 10.1016/B978-0-12-370491-7.00026-X

[7] Balland E, Dam J, Langlet F, Caron E, Steculorum S, Messina A et al (2014) Hypothalamic tanycytes are an ERK-gated conduit for leptin into the brain. Cell Metab 19(2):293–301. 10.1016/j.cmet.2013.12.015 PubMed PMID: 24506870; PubMed Central PMCID: PMC3936883 PMC3936883 24506870

[8] Mazucanti CH, Liu QR, Lang D, Huang N, O’Connell JF, Camandola S et al (2019) Release of insulin produced by the choroid plexis is regulated by serotonergic signaling. JCI Insight 4(23):e131682. 10.1172/jci.insight.131682 PMC6962018 31647782

[9] Enwere E, Shingo T, Gregg C, Fujikawa H, Ohta S, Weiss S (2004) Aging results in reduced epidermal growth factor receptor signaling, diminished olfactory neurogenesis, and deficits in fine olfactory discrimination. J Neurosci Off J Soc Neurosci 24(38):38. 10.1523/JNEUROSCI.2751-042004 PubMed PMID: 15385618; PubMed Central PMCID: PMC6729689 PMC6729689 15385618

[10] Greenwood S, Swetloff A, Wade AM, Terasaki T, Ferretti P (2008) Fgf2 is expressed in human and murine embryonic choroid plexus and affects choroid plexus epithelial cell behaviour. Cerebrospinal Fluid Res 5:20. 10.1186/1743-8454-5-20 PubMed PMID: 19114013; PubMed Central PMCID: PMC2639535 PMC2639535 19114013

[11] Thouvenot E, Lafon-Cazal M, Demettre E, Jouin P, Bockaert J, Marin P (2006) The proteomic analysis of mouse choroid plexus secretome reveals a high protein secretion capacity of choroidal epithelial cells. Proteomics 6(22):5941–5952. 10.1002/pmic.200600096 PubMed PMID: 17051638 17051638

[12] Marques F, Sousa JC, Coppola G, Gao F, Puga R, Brentani H et al (2011) Transcriptome signature of the adult mouse choroid plexus. Fluids Barriers CNS 8(1):10. 10.1186/2045-8118-8-10 PubMed PMID: 21349147; PubMed Central PMCID: PMC3042978 PMC3042978 21349147

[13] Mazucanti CH, Kennedy V, Premathilake HU, Doyle ME, Tian J, Liu QR et al (2023) AAV5-mediated manipulation of insulin expression in choroid plexus has long-term metabolic and behavioral consequences. Cell Rep 42(8):8. 10.1016/j.celrep.2023.112903 PubMed PMID: 37515772; PubMed Central PMCID: PMC10529429 PMC10529429 37515772

[14] Agrawal R, Reno CM, Sharma S, Christensen C, Huang Y, Fisher SJ (2021) Insulin action in the brain regulates both central and peripheral functions. Am J Physiol Endocrinol Metab 321(1):E156–E163. 10.1152/ajpendo.00642.2020 PubMed PMID: 34056920; PubMed Central PMCID: PMC8321819 PMC8321819 34056920

[15] Milstein JL, Ferris HA (2021) The brain as an insulin-sensitive metabolic organ. Mol Metab 52:101234. 10.1016/j.molmet.2021.101234 PubMed PMID: 33845179; PubMed Central PMCID: PMC8513144 PMC8513144 33845179

[16] Baskin DG, Brewitt B, Davidson DA, Corp E, Paquette T, Figlewicz DP et al (1986) Quantitative autoradiographic evidence for insulin receptors in the choroid plexus of the rat brain. Diabetes 35(2):246–249. 10.2337/diab.35.2.246 PubMed PMID: 3510931 3510931

[17] Yao Q, Mazucanti CH, Gomes PA, Liu Q, Egan JM (2025) Precise visualization of insulin receptors A and B in murine brain with an RNA in situ hybridization assay. J Vis Exp JoVE (221):e68677. 10.3791/68677 40758680

[18] Ho KH, Candat A, Scarpetta V, Faucourt M, Weill S, Salio C et al (2023) Choroid plexuses carry nodal-like cilia that undergo axoneme regression from early adult stage. Dev Cell 58(23):23. 10.1016/j.devcel.2023.10.003 PubMed PMID: 37890489 37890489

[19] Mao S, Song R, Jin S, Pang S, Jovanovic A, Zimmerman A et al (2025) Multicilia dynamically transduce Sonic Hedgehog signaling to regulate choroid plexus functions. Cell Rep 44(3):115383. 10.1016/j.celrep.2025.115383 PubMed PMID: 40057957 40057957

[20] Banizs B, Komlosi P, Bevensee MO, Schwiebert EM, Bell PD, Yoder BK (2007) Altered pH(i) regulation and Na(+)/HCO3(-) transporter activity in choroid plexus of cilia-defective Tg737(orpk) mutant mouse. Am J Physiol Cell Physiol 292(4):4. 10.1152/ajpcell.00408.2006 PubMed PMID: 17182727 17182727

[21] Li Y, Di C, Song S, Zhang Y, Lu Y, Liao J et al (2023) Choroid plexus mast cells drive tumor-associated hydrocephalus. Cell 186(26):26. 10.1016/j.cell.202311.001 PubMed PMID: 38056463 38056463

[22] Dodd DO, Mechaussier S, Yeyati PL, McPhie F, Anderson JR, Khoo CJ et al (2024) Ciliopathy patient variants reveal organelle-specific functions for TUBB4B in axonemal microtubules. Science 384(6694):6694. 10.1126/science.adf5489 PubMed PMID: 38662826; PubMed Central PMCID: PMC7616230 PMC7616230 38662826

[23] Ishikawa H, Marshall WF (2017) Intraflagellar Transport and Ciliary Dynamics. Cold Spring Harb Perspect Biol 9(3):a021998. 10.1101/cshperspect.a021998 PMC5334256 28249960

[24] Follit JA, Tuft RA, Fogarty KE, Pazour GJ (2006) The intraflagellar transport protein IFT20 is associated with the Golgi complex and is required for cilia assembly. Mol Biol Cell 17(9):9. 10.1091/mbc.e06-02-0133 PubMed PMID: 16775004; PubMed Central PMCID: PMC1593158 PMC1593158 16775004

[25] Wang X, Yin G, Yang Y, Tian X (2025) Ciliary and Non-Ciliary Roles of IFT88 in Development and Diseases. Int J Mol Sci 26(5):5. 10.3390/ijms26052110 PubMed PMID: 40076734; PubMed Central PMCID: PMC11901018 PMC11901018 40076734

[26] Portal C, Rompolas P, Lwigale P, Iomini C (2019) Primary cilia deficiency in neural crest cells models anterior segment dysgenesis in mouse. eLife 8:e52423. 10.7554/eLife.52423 PMC6946567 31845891

[27] Fossat N, Le Greneur C, Béby F, Vincent S, Godement P, Chatelain G et al (2007) A new GFP-tagged line reveals unexpected Otx2 protein localization in retinal photoreceptors. BMC Dev Biol 7(1):122. 10.1186/1471-213X-7-122 PMC2204009 17980036

[28] Barkho BZ, Monuki ES (2015) Proliferation of Cultured Mouse Choroid Plexus Epithelial Cells. PLoS ONE 10(3):3. 10.1371/journal.pone.0121738 PMC4376882 25815836

[29] Schindelin J, Arganda-Carreras I, Frise E, Kaynig V, Longair M, Pietzsch T et al (2012) Fiji: an open-source platform for biological-image analysis. Nat Methods 9(7):676–682. 10.1038/nmeth.2019 PMC3855844 22743772

[30] Choi HMT, Schwarzkopf M, Fornace ME, Acharya A, Artavanis G, Stegmaier J et al (2018) Third-generation in situ hybridization chain reaction: multiplexed, quantitative, sensitive, versatile, robust. Dev Camb Engl 145(12):dev165753. 10.1242/dev.165753 PubMed PMID: 29945988; PubMed Central PMCID: PMC6031405 PMC6031405 29945988

[31] Ho KH, Patrizi A (2021) Assessment of common housekeeping genes as reference for gene expression studies using RT-qPCR in mouse choroid plexus. Sci Rep 11(1):1. 10.1038/s41598-021-82800-5 PMC7870894 33558629

[32] Ruijter JM, Ramakers C, Hoogaars WMH, Karlen Y, Bakker O, Van Den Hoff MJB et al (2009) Amplification efficiency: linking baseline and bias in the analysis of quantitative PCR data. Nucleic Acids Res 37(6):e45–e45. 10.1093/nar/gkp045 PMC2665230 19237396

[33] Lazarevic I, Engelhardt B (2015) Modeling immune functions of the mouse blood–cerebrospinal fluid barrier in vitro: primary rather than immortalized mouse choroid plexus epithelial cells are suited to study immune cell migration across this brain barrier. Fluids Barriers CNS 13(1):1. 10.1186/s12987-016-0027-0 PMC4734852 26833402

[34] Martin AC, Goldstein B (2014) Apical constriction: themes and variations on a cellular mechanism driving morphogenesis. Dev Camb Engl 141(10):1987–1998. 10.1242/dev.102228 PMC4011084 24803648

[35] Uchida Y, Ohtsuki S, Katsukura Y, Ikeda C, Suzuki T, Kamiie J et al (2011) Quantitative targeted absolute proteomics of human blood–brain barrier transporters and receptors. J Neurochem 117(2):2. 10.1111/j.1471-4159.2011.07208.x 21291474

[36] MacAulay N, Keep RF, Zeuthen T (2022) Cerebrospinal fluid production by the choroid plexus: a century of barrier research revisited. Fluids Barriers CNS 19(1):1. 10.1186/s12987-022-00323-1 PubMed PMID: 35317823; PubMed Central PMCID: PMC8941821 PMC8941821 35317823

[37] Deffner F, Gleiser C, Mattheus U, Wagner A, Neckel PH, Fallier-Becker P et al (2022) Aquaporin-4 expression in the human choroid plexus. Cell Mol Life Sci CMLS 79(2):2. 10.1007/s00018-022-04136-1 PubMed PMID: 35072772; PubMed Central PMCID: PMC8785037 PMC8785037 35072772

[38] Takebayashi G, Chiba Y, Wakamatsu K, Murakami R, Miyai Y, Matsumoto K et al (2023) E-Cadherin Is Expressed in Epithelial Cells of the Choroid Plexus in Human and Mouse Brains. Curr Issues Mol Biol 45(10):7813–7826. 10.3390/cimb45100492 PubMed PMID: 37886936; PubMed Central PMCID: PMC10605538 PMC10605538 37886936

[39] Zappaterra MW, Lehtinen MK (2012) The cerebrospinal fluid: regulator of neurogenesis, behavior, and beyond. Cell Mol Life Sci CMLS 69(17):2863–2878. 10.1007/s00018-012-0957-x PubMed PMID: 22415326; PubMed Central PMCID: PMC3856656 PMC3856656 22415326

[40] Stastný F, Rychter Z (1976) Effect of hydrocortisone on the growth of choroid plexus and composition of cerebrospinal fluid in the developing chick embryo. Acta Neurol Scand 53(4):260–274. 10.1111/j.1600-0404.1976.tb04347x PubMed PMID: 1266572 1266572

[41] Gram M, Ekström C, Holmqvist B, Carey G, Wang X, Vallius S et al (2021) Insulin-like growth factor 1 in the preterm rabbit pup: characterization of cerebrovascular maturation following administration of recombinant human insulin-like growth factor 1/insulin-like growth factor 1-binding protein 3. Dev Neurosci 43(5):281–295. 10.1159/000516665 PMC8623584 34218224

[42] Johanson CE, Murphy VA (1990) Acetazolamide and insulin alter choroid plexus epithelial cell [Na+], pH, and volume. Am J Physiol 258(6 Pt 2):F1538–1546. 10.1152/ajprenal.1990.258.6.F1538 PubMed PMID: 2193541 2193541

[43] Christensen ST, Clement CA, Satir P, Pedersen LB (2012) Primary cilia and coordination of receptor tyrosine kinase (RTK) signalling. J Pathol 226(2):172–184. 10.1002/path.3004 PubMed PMID: 21956154; PubMed Central PMCID: PMC4294548 PMC4294548 21956154

[44] Christensen ST, Morthorst SK, Mogensen JB, Pedersen LB (2017) Primary cilia and coordination of receptor tyrosine kinase (RTK) and transforming growth factor β (TGF-β) signaling. Cold Spring Harb Perspect Biol 9(6):a028167 10.1101/cshperspect.a028167 PMC5453389 27638178

[45] Neugebauer JM, Amack JD, Peterson AG, Bisgrove BW, Yost HJ (2009) FGF signalling during embryo development regulates cilia length in diverse epithelia. Nature 458(7238):651–654. 10.1038/nature07753 PubMed PMID: 19242413; PubMed Central PMCID: PMC2688717 PMC2688717 19242413

[46] Kunova Bosakova M, Nita A, Gregor T, Varecha M, Gudernova I, Fafilek B et al (2019) Fibroblast growth factor receptor influences primary cilium length through an interaction with intestinal cell kinase. Proc Natl Acad Sci U S A 116(10):4316–4325. 10.1073/pnas.1800338116 PubMed PMID: 30782830; PubMed Central PMCID: PMC6410813 PMC6410813 30782830

[47] Anvarian Z, Mykytyn K, Mukhopadhyay S, Pedersen LB, Christensen ST (2019) Cellular signalling by primary cilia in development, organ function and disease. Nat Rev Nephrol 15(4):199–219. 10.1038/s41581-019-0116-9 PubMed PMID: 30733609; PubMed Central PMCID: PMC6426138 PMC6426138 30733609

[48] Gerdes JM, Christou-Savina S, Xiong Y, Moede T, Moruzzi N, Karlsson-Edlund P et al (2014) Ciliary dysfunction impairs beta-cell insulin secretion and promotes development of type 2 diabetes in rodents. Nat Commun 5(1):5308. 10.1038/ncomms6308 25374274

[49] Cho JH, Li ZA, Zhu L, Muegge BD, Roseman HF, Lee EY et al (2022) Islet primary cilia motility controls insulin secretion. Sci Adv 8(38):eabq8486. 10.1126/sciadv.abq8486 PMC9506710 36149960

[50] Kiesel P, Alvarez Viar G, Tsoy N, Maraspini R, Gorilak P, Varga V et al (2020) The molecular structure of mammalian primary cilia revealed by cryo-electron tomography. Nat Struct Mol Biol 27(12):1115–1124. 10.1038/s41594-020-0507-4 PubMed PMID: 32989303; PubMed Central PMCID: PMC7610599 PMC7610599 32989303

[51] Choi E, Duan C, Bai XC (2025) Regulation and function of insulin and insulin-like growth factor receptor signalling. Nat Rev Mol Cell Biol 26(7):558–580. 10.1038/s41580-025-00826-3 PubMed PMID: 39930003; PubMed Central PMCID: PMC12631569 PMC12631569 39930003

[52] Nagao H, Cai W, Wewer Albrechtsen NJ, Steger M, Batista TM, Pan H et al (2021) Distinct signaling by insulin and IGF-1 receptors and their extra- and intracellular domains. Proc Natl Acad Sci 118(17):e2019474118. 10.1073/pnas.2019474118 PMC8092608 33879610

[53] Réthelyi JM, Vincze K, Schall D, Glennon J, Berkel S (2023) The role of insulin/IGF1 signalling in neurodevelopmental and neuropsychiatric disorders – Evidence from human neuronal cell models. Neurosci Biobehav Rev 153:105330. 10.1016/j.neubiorev.2023.105330 37516219

[54] Rudick VL, Rudick MJ, Brun-Zinkernagel AM (1993) Amplification of the Golgi complex in MDCK cells secreting human growth hormone. J Cell Sci 104(2):509–520. 10.1242/jcs.104.2.509 8505376

[55] Sin ATW, Harrison RE (2016) Growth of the Mammalian Golgi Apparatus during Interphase. Mol Cell Biol 36(18):2344–2359. 10.1016/j.neubiorev.2023.105330 PMC5007790 27325676

[56] Haeusler RA, McGraw TE, Accili D (2018) Biochemical and cellular properties of insulin receptor signalling. Nat Rev Mol Cell Biol 19(1):31–44. 10.1038/nrm.2017.89 PMC5894887 28974775

[57] Le TKC, Dao XD, Nguyen DV, Luu DH, Bui TMH, Le TH et al (2023) Insulin signaling and its application. Front Endocrinol 14:1226655. 10.3389/fendo.2023.1226655 PubMed PMID: 37664840; PubMed Central PMCID: PMC10469844 PMC10469844 37664840

[58] Banks WA, Owen JB, Erickson MA (2012) Insulin in the brain: There and back again. Pharmacol Ther 136(1):82–93. 10.1016/j.pharmthera.2012.07.006 PMC4134675 22820012

[59] De Meyts P (2004) Insulin and its receptor: structure, function and evolution. BioEssays News Rev Mol Cell Dev Biol 26(12):1351–1362. 10.1002/bies.20151 PubMed PMID: 15551269 15551269

[60] Wallum BJ, Taborsky GJ, Porte D, Figlewicz DP, Jacobson L, Beard JC et al (1987) Cerebrospinal fluid insulin levels increase during intravenous insulin infusions in man. J Clin Endocrinol Metab 64(1):190–194. 10.1210/jcem-64-1-190 PubMed PMID: 3536982 3536982

[61] Geijselaers SLC, Aalten P, Ramakers IHGB, De Deyn PP, Heijboer AC, Koek HL et al (2018) Association of Cerebrospinal Fluid (CSF) Insulin with Cognitive Performance and CSF Biomarkers of Alzheimer’s Disease. J Alzheimers Dis JAD 61(1):309–320. 10.3233/JAD-170522 PubMed PMID: 29154275; PubMed Central PMCID: PMC5734123 PMC5734123 29154275

[62] Israel PA, Park CR, Schwartz MW, Green PK, Sipols AJ, Woods SC et al (1993) Effect of diet-induced obesity and experimental hyperinsulinemia on insulin uptake into CSF of the rat. Brain Res Bull 30(5–6):571–575. 10.1016/0361-9230(93)90084-O 8457906

[63] Heni M, Schöpfer P, Peter A, Sartorius T, Fritsche A, Synofzik M et al (2014) Evidence for altered transport of insulin across the blood–brain barrier in insulin-resistant humans. Acta Diabetol 51(4):679–681. 10.1007/s00592-013-0546-y 24370925

[64] Nameni G, Farhangi MA, Hajiluian G, Shahabi P, Abbasi MM (2017) Insulin deficiency: A possible link between obesity and cognitive function. Int J Dev Neurosci 59(1):15–20. 10.1016/j.ijdevneu.2017.02.008 28274759

[65] Ávalos Y, Hernández-Cáceres MP, Lagos P, Pinto-Nuñez D, Rivera P, Burgos P et al (2022) Palmitic acid control of ciliogenesis modulates insulin signaling in hypothalamic neurons through an autophagy-dependent mechanism. Cell Death Dis 13(7):7. 10.1038/s41419-022-05109-9 PubMed PMID: 35902579; PubMed Central PMCID: PMC9334645 PMC9334645 35902579

