# Supplementary material for: Insulin-mediated ciliary remodeling in the choroid plexus epithelium
Source: Cell Mol Life Sci. 2026 Oct 15;83(1):382. doi: 10.1007/s00018-026-06486-6 (PMC13675167; doi:10.1007/s00018-026-06486-6)
Supplement: Supplementary file 1 [file 18_2026_6486_MOESM1_ESM.docx]

**Cellular and Molecular Life Sciences**

**Supplementary materials to:**

**Insulin-mediated ciliary remodeling in the choroid plexus epithelium**

Marleen Trapp^1,§^, L. Aurica Ritter^1^, Greta Döking^2^, James Cleland^2^, Anthony D. Hill^3^, Annarita Patrizi^1,3*^

^1^Schaller Research Group, German Cancer Research Center (DKFZ), Heidelberg, Germany;

^2^Division of Regulatory Genomics and Cancer Evolution, German Cancer Research Center (DKFZ), Heidelberg, Germany;

^3^Department of Neurobiology, Interdisciplinary Centre for Neurosciences (IZN), Heidelberg University, Heidelberg, Germany;

^§^current address: Division of Translational Pediatric Sarcoma Research and Division of Clinical Cooperation Unit Virotherapy, German Cancer Research Center (DKFZ), Heidelberg, Germany.

*Corresponding author:

Annarita Patrizi, PhD

[patrizi@nbio.uni-heidelberg.de](mailto:patrizi@nbio.uni-heidelberg.de)

The authors declare no potential conflict of interest.

**Supplementary Figure 1: Establishment of primary cell culture from mouse choroid plexus epithelial cells and growth factor treatment. a,** Representative brightfield image of primary choroid plexus epithelial cell culture (pCPEC) at day *in vitro* (div) 6; **b,** Representative confocal images of pCPEC stained against cytokeratin (green) at div6; **c,** Quantification of the percentage (%) of cytokeratin positive (^+^) cells at div6, *n* = 9 images, from 2 biological replicates; **d,** Representative images of EdU^+^ pCPECs (magenta) at div6; **e,** Quantification of % of EdU^+^ cells at div6, *n* = 20 images, from 3 biological replicates; **f,** Fold change of total cell density of pCPECs treated with FGF, EGF, HC, INS and IGF-1 compared to untreated cells, *n* = 10-15 images, from 3 biological replicates; **g**, Fold change of nuclei surface area of pCPECs treated with FGF, EGF, HC, INS and IGF-1 compared to untreated cells, *n* = 10-15 images, from 3 biological replicates; **h,** Representative images of cilia stages (single ciliated (sc) cells, multiciliated disengaged cells (mcd) and multiciliated cells (mcc) in pCPEC stained against γ-tubulin (green), ARL13B (magenta) and ZO-1 (grey) at div6; **i,** Quantification of cilia types at div6, *n* = 14 images, from 3 biological replicates; **j,k,** Fold change of the number of pCPEC axonemes in non-mcc (**j**) and mcc cells (**k**), *n* = 15-50 cells per treatment, from 3 biological replicates; **l,** Fold change of axoneme length of sc (left) and mcd (right) cells, *n* = 10-20 cilia, from 3 biological replicates; **m,** Fold change of apical cell area in pCPEC at different cilia organizational stages after treatment with FGF, EGF, HC, INS and IGF-1 compared to untreated cells., *n* = 6-20 cells per treatment, from 3 biological replicates; Scale bar 100 µm (**a**, **b**, **d**), 10 µm (**h**). Black line: median; Kruskal-Wallis test followed by Duns multiple comparison (**f, g**, **j k**), Two-Way ANOVA followed by Šidák`s multiple comparisons test, (**l**, **m**); ****p < 0.0001. INS, Insulin; IGF-1, insulin growth factor 1; FGF, Fibroblast growth factor; EGF: Epidermal growth factor; HC: Hydrocortisone.

**Supplementary Figure 2: Atlases of choroid plexus at embryonic and adult stages.** **a,** UMAP distribution of single nuclei RNA-sequencing data colored by subtype identity. E., embryonic; A., adult. **b,** UMAP expression of *Insr* mRNA in whole choroid plexus cells at embryonic and adult stages.

**Supplementary Figure 3: Cellular and cilia features in short and acute insulin treatments. a,b,** Fold change of the percentage (%) of non-multiciliated (non-mcc) (**a**) and non-ciliated (**b**) primary choroid plexus epithelial cells (pCPECs) treated with insulin for 24 or 48 hours (h), *n* = 10-15 images, from 3 biological replicates; **c,d,** Fold change of the axoneme number per cell in non-mcc (**c**) and mcc (**d**) pCPEC, *n* = 40-90, from 3 biological replicates; **e,** Fold change of the axoneme length in non-mcc pCPECs, *n* = 370-400 cilia, from 3 biological replicates; **f,g,** Fold change of the % of non-mcc (**f**) and non-ciliated (**g**) pCPECs treated with insulin for the first 24h or 48h followed by 5 or 4 days (d) without treatment (24h+5d; 48h+4d, respectively), *n*=10-15 images, from 3 biological replicates; **h,i,** Fold change of the axoneme number of per cell in non-mcc (**h**) and mcc (**i**) pCPECs, *n* = 40-70 cells, from 3 biological replicates; **j,** Fold change of the axoneme length in non-mcc treated pCPECs, *n* = 40-70 cilia, from 3 biological replicates. Black dashed line represents chronic insulin condition. Green lines represent median; Kruskal-Wallis test followed by Duns multiple comparison; **p < 0.01, # indicates significance between c-INS and 24h, 48h, 24h+5d, 48h+4d; ^#^p < 0.05, ^###^p < 0.001, ^####^p < 0.0001.
